# Colorectal Cancer Screening using Immunochemical Faecal Occult Blood Testing (iFOBT) in Urban-Poor Communities in Cheras, Malaysia: A Cross-Sectional Study

**DOI:** 10.21315/mjms-09-2024-726

**Published:** 2025-02-28

**Authors:** Muhammad Irfan Abdul Jalal, Ahmad Termidzi Mohd Azhar, Mohd Arman Kamaruddin, Mohd Raziff Alias, Nazihah Abd Jalal, Norliza Ismail, Siok-Fong Chin, Ying-Xian Goh, Noraidatulakma Abdullah, Ismail Sagap, Zairul Azwan Mohd Azman, Azimatun Noor Aizuddin, Azmawati Mohamed Nawi, Nor Halizam Ismail, Rahman Jamal, Nor Azian Abdul Murad

**Affiliations:** 1 UKM Medical Molecular Biology Institute, Universiti Kebangsaan Malaysia, Cheras, Kuala Lumpur, Malaysia; 2 Hospital Tunku Ampuan Besar Tuanku Aishah Rohani, Universiti Kebangsaan Malaysia, Cheras, Kuala Lumpur, Malaysia; 3 Hospital Canselor Tuanku Muhriz, Universiti Kebangsaan Malaysia, Cheras, Kuala Lumpur, Malaysia; 4 Department of Public Health Medicine, Faculty of Medicine, Universiti Kebangsaan Malaysia, Cheras, Kuala Lumpur, Malaysia; 5 Department of Health and Environment, Dewan Bandaraya Kuala Lumpur, Cheras, Kuala Lumpur, Malaysia

**Keywords:** colonoscopy, colorectal cancer, early screening, faecal occult blood test, urban-poor

## Abstract

**Background:**

Colorectal cancer (CRC) is one of the most common cancers globally, with the immunochemical faecal occult blood test (iFOBT) frequently used for population level screening. This study evaluated CRC screening uptake among urban-poor individuals aged 40–65, assessed their knowledge of CRC risk factors and symptoms before and after an educational programme, and identified determinants of polyps and CRC within this group.

**Methods:**

A cross-sectional study recruited 577 individuals from seven People’s Residential Project (PPR) areas in Cheras, Kuala Lumpur and Malaysia Madani Carnival between March 2022 and July 2023. Inclusion criteria were age 40–65 and smartphone ownership, excluding those with CRC history, acute gastritis, inflammatory bowel disease, or recent CRC screening. The iFOBT was administered, followed by questionnaires and educational talks. A follow-up questionnaire was conducted via phone two weeks post-programme.

**Results:**

Overall, 321 participants fulfilled the eligibility criteria. Most iFOBT-positive participants were in their 50s (median [interquartile range, IQR]: 56 [16]), female (65%), 86.3% non-smokers, and 62.5% with moderate CRC risk based on the Asia Pacific Colorectal Screening (APCS) Score, showing no significant differences from iFOBT-negative participants. Among the 267 who returned iFOBT kits, 30.0% tested positive, with 28.8% undergoing colonoscopy. Polyps and CRC were detected in 21.74% and 4.35% of the participants, respectively. The mean knowledge score on CRC symptoms was significantly lower post-programme, with no significant change in awareness of CRC risk factors.

**Conclusion:**

Detection rates for polyps and CRC are low. Awareness of CRC symptoms is higher pre-screening than post-screening, highlighting challenges in conducting CRC education in urban-poor communities.

## Introduction

Colorectal cancer (CRC) is the third most common cancer for both men (1,069,446 new cases) and women (856,979 new cases) worldwide in 2022, and it ranks as the second leading cause of cancer deaths globally (1). There has been a rapid increase in the incidence of CRC in many Asian countries during the past few decades. The lifetime risks of CRC in Malaysian men and women have escalated in 2017–2021 to one in 44 and one in 62 individuals, compared to one in 56 and one in 76 individuals reported between 2012–2016 (2). CRC is the most common cancer among men and the second most common among women in Malaysia (2–3). Although CRC screening is a well-established practice for average and high-risk individuals in developed countries, it has not been widely implemented in most developing countries (4). The inadequate uptake and execution of CRC screening in many countries are linked to limited resources, insufficient support from health authorities, and low public awareness.

Malaysia comprises three major ethnic groups, including Malays (69.4%), Chinese (23.2%), Indian (6.7%), and others (0.7%) (5). To overcome the rise in CRC morbidity and mortality trends, the Ministry of Health Malaysia (MOH) in 2021 have drafted and implemented a National Strategic Plan for Colorectal Cancer (NSPCRC) 2021–2025, which highlights the steps to improve the Current National Pragmatic Response towards CRC screening, treatment and prevention including the use of single immunological faecal occult blood test kit (iFOBT) (3). The plan is to screen for early detection of CRC in asymptomatic individuals in selected health clinics for individuals aged between 50 and 75 years old (3). While early screening is essential for CRC prevention and reducing mortality, research in Malaysia has indicated that lower socioeconomic classes are more likely to be diagnosed at a late or advanced stage of CRC and have poorer survival rates (6). On the other hand, studies in the UK have shown that higher socioeconomic groups are more likely to participate in screening (7–8). Besides, a recent study identified that the response and favourable iFOBT test rates were 79.6% and 13.1% among the healthy volunteer-Malaysian population in urban areas (9). The overall CRC detection rate was 0.3%, while the colorectal neoplasia detection rate (colorectal cancer and colorectal polyps) was 2.3%. Most participants with positive iFOBT results are at high-risk for CRC (7). However, previous research has not sufficiently explored the value of screening asymptomatic urban-poor individuals aged 40 years old to 65 years old. This evidential gap provides the impetus for this research focusing on the urban-poor community, defined as households in the bottom 40% (B40) of income earners according to the Malaysian Department of Statistics’ thresholds (10).

Early detection and treatment of CRC will result in a substantial reduction in treatment costs and mortality rates. The cost of treating the advanced stage of CRC is 1.8 to 2.5-fold higher than early-stage cancer (6, 11). Studies showed the declining trend of CRC incidence and mortality rates in highly developed countries, including the United States, Canada, and Northern Europe, which might be attributed to effective CRC screening programmes (12–13). Although colonoscopy is the gold standard and most effective CRC screening tool, it is an expensive and invasive method that requires a skilled healthcare specialist (14). A more straightforward, quick and non-invasive colorectal cancer screening using the iFOBT is more suitable at the population level. Although it is not superior to colonoscopy, the effectiveness of iFOBT in detecting CRC and reducing mortality is well-established. A 30-year follow-up study using iFOBT screening showed a long-term mortality reduction of 32% for the annual screening and 18% for biennial screening (15). The long-term sustained effect of CRC mortality reduction is mainly attributed to the polypectomy performed due to the iFOBT screening programme (15).

The objectives of this project were to assess the level of CRC screening uptake among poor urban groups aged 40 to 65, evaluate the knowledge and awareness of CRC risk factors and symptoms in the screened population before and after the CRC awareness programme, and finally, identify the determinants of polyps and colorectal cancer in this vulnerable poor urban population.

## Methods

### Study Design and Eligibility Criteria for Subject Recruitment

The Higher Institution Centre of Excellence – UKM Medical Molecular Biology Institute (HiCOE-UMBI) Colorectal Cancer Awareness and Screening programme is a cross-sectional study conducted from March 2022 to July 2023, targeting seven urban-poor areas in Cheras, located in southern Kuala Lumpur, Malaysia: People’s Residential Programme/ Program Perumahan Rakyat (PPR) Desa Tun Razak, PPR Flat Sri Kota, PPR Sri Johor, PPR Sri Labuan, PPR Sri Sabah, PPR Taman Ikan Emas, and PPR Taman Mulia. Participants were also recruited from Karnival Madani Sihat, a national health carnival in Malaysia.

Eligible participants met the following criteria: 1) B40 income group (bottom 40% of household incomes) aged 40–65 years living in the specified PPR areas of Cheras; 2) provided consent; and 3) owned a smartphone. The lower age limit for screening, compared to the Malaysian Clinical Practice Guideline (CPG) on Colorectal Carcinoma Screening (16), was justified by three reasons: i) screening for individuals 40+ years is cost-effective and life-saving (17); ii) this policy is adopted in countries like the UAE and Japan (18); iii) increasing rates of CRC in younger adults globally (19). Exclusions included those with a history of CRC, prior CRC screening, inflammatory bowel disease, or acute gastritis in the past two years. B40 households were defined as having a monthly income below RM 5,250.00 (10). A purposive sampling method was used due to the limited number of eligible participants.

### Ethics Approval

This study was approved by the Medical Research Ethics Committee, Universiti Kebangsaan Malaysia (UKM) on 24 December 2021 (Ref: UKM PPI/111/8/JEP-2021-841). Informed consent was obtained from all participants, and the study adhered to the Declaration of Helsinki.

### A Priori Sample Size Calculation

The sample size was calculated solely to estimate the iFOBT compliance rate due to limited data on the required parameter estimates in the literature for calculating the sample size for other study objectives. A single-proportion formula was implemented via a web-based calculator (20–21). Prior research by Abdullah et al. estimated the proportion of participants returning iFOBT kits at 0.793 (9). The precision (δ) was set at 5%, with a 95% confidence level. This resulted in a required sample size of 253 subjects without dropouts. Accounting for a 40% dropout rate, a total of 422 participants were needed to meet the study objectives.

### Interactive CRC Awareness Talk

Interactive CRC Awareness Talks, conducted in the national language, were held in all targeted communities to raise awareness about CRC, focusing on healthy diet and lifestyle, delivered by a clinician who was also a study investigator. Pre-event activities included strategic meetings with key community leaders and project partners, as well as displaying campaign posters and banners in prominent locations. Registration involved detailed interviews and history-taking by trained personnel, gathering participants’ demographic, medical, and lifestyle information. Registered participants received tutorial-style instructions on using and returning the faecal immunohistochemical test (FIT) kit. During recruitment, participants completed pre-test questionnaires on CRC awareness and risk behaviours. The importance of the screening test and its use was explained during registration and the awareness talk, which also covered CRC risk factors, management options, and follow-up diagnostic tests like colonoscopy and radiological imaging for cancer staging.

### Faecal Occult Blood Test for CRC Screening

Each participant received two iFOBT kits (OC-Light S Fit REF V-PC100, Eiken, Japan) and was required to return both within 5 days at designated PPR locations. Research personnel collected and processed the faecal samples according to the protocols by Abdullah et al. (9). Two weeks post-event, CRC awareness and risk behaviour were assessed through questionnaires via Google Form.

Participants with negative iFOBT results were notified by mail and advised to undergo biennial iFOBT testing. Those with positive results were contacted by phone and referred for confirmatory colonoscopy and biopsy at Hospital Canselor Tuanku Muhriz (HCTM), UKM. To boost colonoscopy uptake, a medically trained researcher conducted door-to-door visits to explain results, address concerns, and encourage attendance at HCTM. Personalised discussions using audiovisual aids were also held with iFOBT-positive participants, covering the colonoscopy process, its rationale, and potential therapeutic options if polyps or CRC were found. Participants refusing referral were advised to have annual iFOBT testing.

Participants were followed up every 6 months to update demographic information, including current addresses and CRC screening status. Those with positive colonoscopy findings were referred to the HCTM Surgical clinic for further treatment, while participants with negative colonoscopy results were advised to repeat iFOBT screening every five years. The study flow is detailed in Appendix 1, aligning with the MOH National Guidelines for Patient Navigation Process Flow (3). Reporting followed the STROBE guideline for transparency in cross-sectional studies (22).

### Data Analysis

For each study variable, relevant thresholds were chosen to categorise continuous variables. For instance, body mass index (BMI) was categorised based on the Malaysian Guidelines for the Management of Obesity published in 2023: < 18.5 kg/m^2^ (Underweight); 18.5 kg/ m^2^–22.9 kg/m^2^ (Normal); 23.0 kg/m^2^–27.4 kg/ m^2^ (Overweight); and 27.5 kg/m^2^ (Obese) (23). A positive family history of CRC was defined as any first-degree or two second-degree relatives with a history of CRC before the age of 40 (24). The Asia Pacific Colorectal Screening (APCS) score, a risk-stratification scoring system based on routinely collected clinical variables for identifying subjects at high-risk of CRC, for each participant was calculated as follows: i) Age (< 50 years old = 0; 50 years old–69 years old = 2; 70 years old and above = 3); ii) Gender (Female = 0; Male = 1); iii) Smoking status (No = 0; Present smokers = 2); iv) Family history of CRC (Absent = 0; Present = 2). The scores were then totalled and further divided into three-tier categories: i) low risk (0–1 point), ii) intermediate-risk (2–3 points), and iii) high-risk (4–7 points) (25). The APCS scores have been validated in many Asian populations and demonstrated high sensitivity and specificity in detecting patients with advanced colorectal cancer (26–27).

The baseline characteristics of the participants were described in mean (SD) or median (interquartile range, IQR) for non-normally distributed variables and in frequency (percentage) for categorical variables. Multiple imputations for missing data were considered only if the missing rate was between 5% and 40% (28). A paired *t*-test was performed to compare the differences in the knowledge level of the participants before and after the CRC awareness programme, and a chi-squared test (or Fisher’s exact test if over 20% of the cells in a contingency table have an expected count of less than 5) was used to test the associations between the sociodemographic profiles of the study participants and iFOBT results. The paired *t*-test assumption of normality in pre-CRC and post-CRC programme differences in CRC risk factors and symptom scores was objectively assessed using the Shapiro-Wilks test and Fisher’s skewness coefficient (normality threshold ±3.29 for 50 ≤ *n* < 300) (29).

Simple logistic regression was performed to identify possible determinants of colorectal polyps and CRC. Clinically and theoretically important variables were selected for multivariable model building using hierarchical or theory-driven approaches, regarded as more suitable than automated stepwise methods (30–31). Two logistic regression models were created for each outcome to address multicollinearity between APCS score groups and its components (gender, age, family history of CRC, and smoking status): (i) a model including all clinically important predictors without APCS score groups and (ii) a model with APCS score groups plus other predictors not part of APCS, such as diabetes status, ethnicity, and BMI.

Predictor significance was evaluated with *p*-values from the Wald statistic (for continuous or binary predictors) or the partial likelihood ratio test (for categorical predictors with multiple levels) (32). Effect modifiers were assessed through interaction terms created for medically sensible interactions, and multicollinearity was checked with inter-predictor correlations above 0.70 (33).

Model calibration and discrimination were assessed using the Hosmer-Lemeshow test (*p* > 0.05 for adequate calibration), accuracy (> 80%), and AUC (≥ 70% for adequate discrimination) (32). Influential observations were identified with difference in betas (DFBETA) (threshold: 1); observations with implausible values were excluded, while those with plausible values were retained (32). Results were presented as crude and adjusted odds ratios (ORadj) with 95% confidence intervals, and no sensitivity analysis was performed as it was not pre-planned.

All statistical procedures were performed using SPSS Version 29 (IBM, Armonk, NY, USA, 2023).

### Results

**Figure 1:**
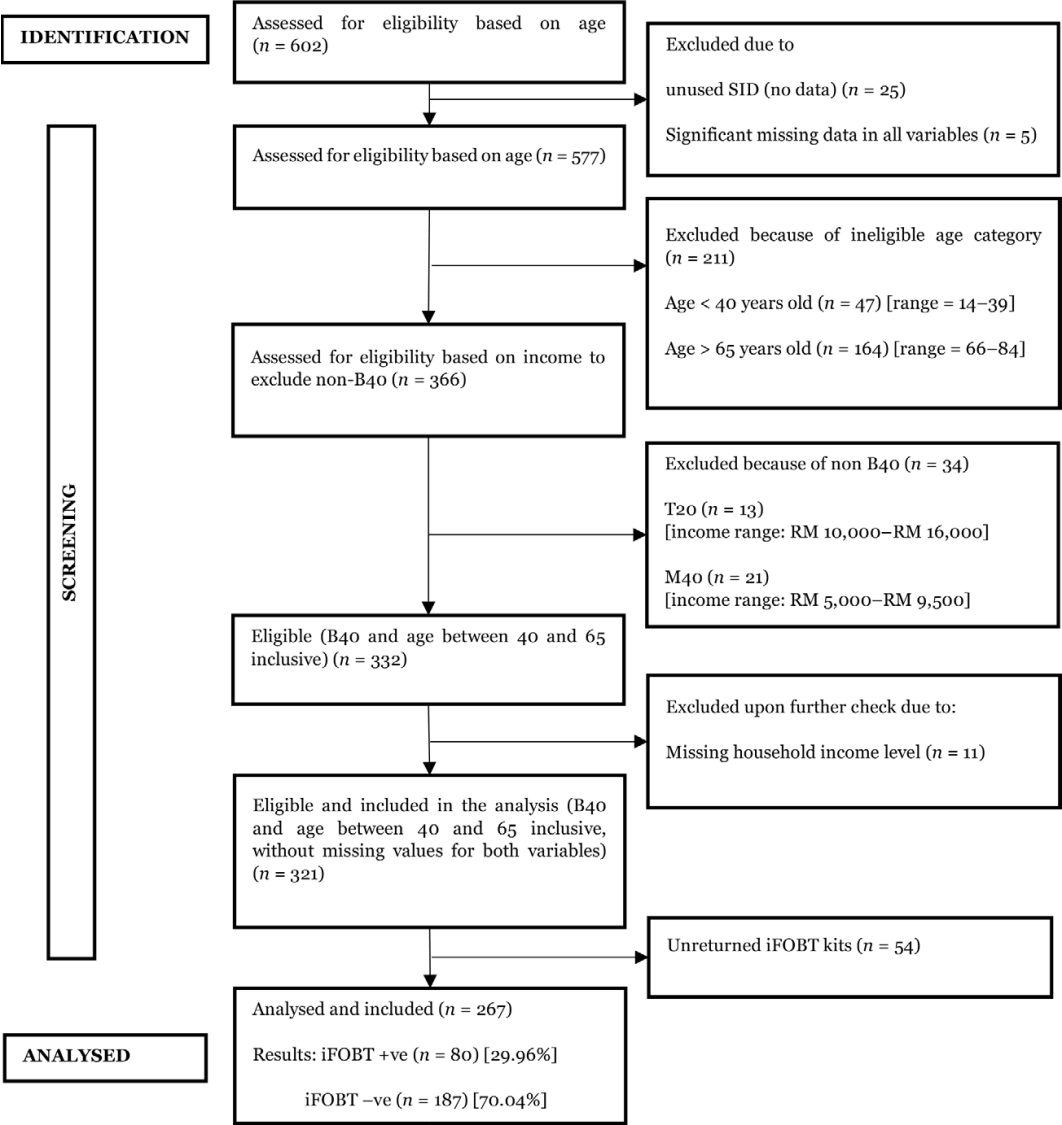
The STROBE chart summarises the flow of participants in this study

**Figure 2:**
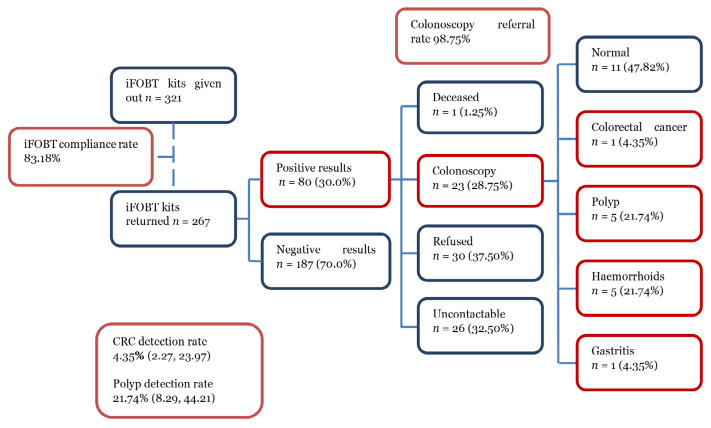
Summary of iFOBT screening

In total, 577 participants agreed to participate in the screening programme, and 256 were excluded due to unrelated age (under 40 years or more than 65 years old), non-B40 group, and missing household income (Figure 1). In total, 321 participants were included in this study. Of these, 267 participants returned the kits, resulting in an iFOBT compliance rate of 83.2% (Appendix 2). Among this set of study participants, 80 (30.0%) subjects tested positive for faecal occult blood, and 187 were negative. Out of these iFOBT-positive subjects, 79 (98.75%) subjects were referred for colonoscopy. However, only 23 participants willingly underwent colonoscopy, resulting in a colonoscopy uptake rate of 28.8%. Five subjects from this subset of participants were discovered to have benign polyps. Based on the colonoscopy findings, only one individual was confirmed to have early-stage, well-differentiated CRC. Hence, the polyp and CRC detection rates were 21.74% (95% CI 8.29%; 44.21%) and 4.35% (95% CI 2.27%; 23.97%), respectively. Figure 2 summarises the relevant metrics for the iFOBT screening.

### Baseline Characteristics of Study Participants

The majority of those with positive iFOBT results were female (65%), of Malay ethnicity (80%), had no family history of CRC (93.8%), non-smokers (86.3%) and non-diabetics (65%). Around four-fifths of them were either overweight or obese. No significant associations were observed between all sociodemographic status of the study participants and iFOBT results. The details of baseline sociodemographic profiles of the participants are listed in Table 1.

The patient with CRC is a 65 year old Malay gentleman who is obese (BMI = 27.9 kg/m^2^) and a smoker with a personal history of diabetes. This patient had no family background of colorectal cancer. Five individuals were identified as having polyps. Patient 001 is a Chinese gentleman, age 62 years old, with an overweight BMI (23.8 kg/m^2^), no family history of CRC, is a non-smoker and does not have diabetes. Patient 002 is an Indian female, 48 years old, obese (BMI = 31.5 kg/m^2^) without a family history of CRC, non-smoker and does not have diabetes. Patient 003 is a Malay female, 61 years old, obese (BMI = 41.8 kg/m^2^), non-smoker and has no family history of CRC and personal history of diabetes. Patient 004 is a Malay male, 61 years old, with diabetes, no family history related to CRC, non-smoker and is obese (BMI = 29.1 kg/ m^2^). Patient 005 is also a Malay male, 58 years old, overweight (BMI = 25.8 kg/m^2^), without a family history of CRC, non-smoker and does not have diabetes.

Five individuals with haemorrhoids identified as patient 006 is a 55 year old Malay female, obese (BMI = 31.2 kg/m^2^) with no family history of CRC, non-smoker and does not have diabetes. Patient 007 is a 64 year old Malay female, obese (BMI = 28.1 kg/m^2^), without a family history of CRC, non-smoker, but had diabetes. Patient 008 is an Indian female, 42 years old, without a family history of CRC, non-smoker, but has diabetes and is obese (BMI = 27.6 kg/m^2^). Patient 009 is a 65 year old Malay gentleman without a family history of CRC, a non-smoker but has diabetes and is overweight (BMI = 25.5 kg/m^2^). The last patient, 010, is a 41 year old Chinese female, obese (BMI = 38.7 kg/ m^2^) with a family history of CRC but is a non-smoker and does not have diabetes.

### Clinicodemographic Determinants of Polyps and CRC

Based on simple and multiple logistic regression analyses (Appendix 3), no significant sociodemographic determinant was associated with colorectal polyps’ occurrence. Similar results were also found for CRC (Appendix 4). For multivariable model building, we decided all predictors to be clinically and biologically relevant to colorectal polyps and CRC development and therefore included them in the logistic regression model. No evidence of multicollinearity and significant statistical interactions were found (all possible two-way statistical interactions were checked since they were deemed theoretically feasible). All models demonstrated satisfactory discriminative and calibrative performances (Appendixes 3 and 4) and no influential observations were detected, as evidenced by DFBETA values within the thresholds of ±1.

### Comparisons of Clinicodemographic Profile in Individuals with and without Polyps

Table 2 shows no significant difference in sociodemographic and clinical profiles between participants diagnosed with polyps and without polyps. In those with polyps, the histological findings were tubulovillous adenoma with low-grade dysplasia (*n* = 2, 40%), hyperplastic polyps (*n* = 2, 40%), and tubular adenoma with low-grade dysplasia (*n* = 1, 20%). Besides, the solitary CRC patient discovered a malignant sessile polyp with well-differentiated adenocarcinomatous features and invasion of the middle third of the submucosal layer (Sm2, Kikuchi classification).

### Pre- and Post-programme CRC Awareness

Out of 267 participants, only 190 study participants completed the questionnaires both before and after the CRC awareness programme. Paradoxically, the mean scores of the knowledge levels of study participants about the CRC risk factors and symptoms decreased after the CRC awareness programme, albeit only CRC symptoms and signs mean scores exhibited statistically significant differences. Full results are presented in Table 3.

## Discussion

This study suggests that iFOBT is a useful and inexpensive screening tool for early CRC detection, evidenced by identifying five polyps and one early-stage CRC in urban-poor communities. Despite this, the CRC and polyp detection rates were low, and the high false-positive rate of iFOBT is concerning, though specific causes remain unknown due to the lack of further diagnostic tests like esophagogastroduodenoscopy (OGDS) or enteroscopy performed in our study participants. Therefore, different screening modalities are warranted (35–36). The return rate of iFOBT kits (83.2%) was similar to previous findings (9, 35), indicating satisfactory compliance. However, further research is needed to assess the predictive value of the APCS scoring system for selecting high-risk subgroups to improve screening efficiency.

In comparison to Schliemann et al.’s home-based CRC screening in Malaysia (37), which had a 52% participation rate and 42% completion rate of the iFOBT test, our study showed higher iFOBT kit return rates (83.2%), though colonoscopy uptake remained low at 28.8%. Out of 80 positive iFOBT results, only 23 participants (28.8%) underwent a follow-up colonoscopy. According to the European guideline for quality assurance in CRC screening and diagnosis, the lowest acceptable screening uptake is 45% (38). Considering the large number of B40 households in each PPR in Kuala Lumpur (average family size of five, 1,455 individuals for each PPR block housing 317 dwelling units (39), our small sample size indicates a low participation rate for iFOBT screening in these urban-poor communities due to three reasons: i) discomfort with stool collection; ii) anxiety over positive results; and iii) insufficient awareness about CRC (40–41). Although participants received awareness talks from a clinician, limited exposure through media and education may have hindered engagement, emphasising the need for broader public awareness and culturally sensitive materials.

The low colonoscopy uptake rate (28.75%) reflects challenges like decreased CRC knowledge two weeks after the awareness programme, fear of a diagnosis, and financial concerns (42). Additionally, the COVID-19 pandemic’s disruption and negative perceptions of iFOBT among low-income participants further impeded participation (43). An effective CRC screening programme should address treatment costs and improve awareness efforts to increase colonoscopy uptake.

The single CRC patient underwent successful laparoscopic anterior resection and continues annual surveillance for recurrence. Participants with high-risk polyps are monitored with colonoscopy every three years, following international guidelines (44–46). These findings underscore the role of early detection in reducing CRC morbidity in low-income populations (47).

Although 59.2% of participants completed both pre- and post-programme questionnaires, no significant differences in knowledge scores were found. This might be due to the lengthy questionnaire, conducting post-tests via phone rather than in-person, and participant fatigue. Future studies should conduct surveys face-to-face for better communication and increased engagement.

The cross-sectional nature of this study limits the assessment of long-term CRC screening benefits. Using Google Forms for post-programme evaluations may have impacted the accuracy of measuring knowledge retention. The small sample size reduced the power to detect other outcomes, though it was sufficient for iFOBT compliance estimation. Moreover, the results cannot be generalised to the B40 population in other Malaysian states, as recruitment was limited to PPRs in Cheras, Kuala Lumpur. Tailored CRC screening programmes using affordable FIT kits, combined with targeted engagement strategies like door-to-door campaigns and patient support are critical for successful implementation. Ongoing efforts in educating urban-poor communities remain essential, as early detection can significantly impact outcomes.

## Conclusion

Among 23 participants who underwent colonoscopy, one CRC, five colorectal polyps, and five haemorrhoids were detected, with all referred for further treatment, highlighting its role as an affordable and useful screening tool for early CRC detection. Nevertheless, iFOBT still demonstrated low detection rates for individuals at high-risk of CRC or polyps with potential malignant transformation in urban-poor communities. Poor awareness of CRC risk factors and symptoms likely contributed to low colonoscopy uptake, suggesting that alternative screening strategies are needed to reduce premature CRC-related deaths in these communities.

## Figures and Tables

**Table 1:** Sociodemographic characteristics of participants who returned their iFOBT test kits, stratified by their iFOBT results (*n* = 267)

Characteristics		iFOBT results		*P*-value

		Positive (*n* = 80)	Negative (*n* = 187)	
Age in years (Median [IQR])		56 (16.0)	58 (11.0)	0.195*

Gender	Male	28 (35.0)	62 (33.2)	0.770
	Female	52 (65.0)	125 (66.8)	

Ethnicity	Malay	64 (80.0)	155 (82.9)	0.216
	Chinese	10 (12.5)	11 (5.9)	
	Indian	6 (7.5)	19 (10.2)	
	Others	0 (0.0)	2 (1.0)	

Family history of CRC	Yes	5 (6.2)	14 (7.5)	0.719
	No	75 (93.8)	173 (92.5)	

Smoking status	Yes	11 (13.7)	29 (15.5)	0.712
	No	69 (86.3)	158 (84.5)	

Diabetes status	Yes	28 (35.0)	68 (36.4)	0.832
	No	52 (65.0)	119 (63.6)	

BMI^a^	Underweight	0 (0.0)	2 (1.1)	0.672
	Normal	15 (19.0)	30 (16.3)	
	Overweight	36 (45.6)	78 (42.4)	
	Obese	28 (35.4)	74 (40.2)	

APCS group	Low risk	20 (25.0)	32 (17.1)	0.319
	Moderate-risk	50 (62.5)	127 (67.9)	
	High-risk	10 (12.5)	28 (15.0)	

Notes:

* Based on the Mann-Whitney test, one subject had missing observations in the iFOBT-positive group and three in the iFOBT-negative group.

iFOBT = immunohistochemical faecal occult blood test; IQR = interquartile range; CRC = colorectal cancer; BMI = body mass index; APCS = Asia Pacific Colorectal Screening

**Table 2:** Comparisons of sociodemographic profiles of subjects diagnosed with and without polyps (*n* = 22)+

Variables	Polyps (*n* = 5)	Non-polyps (*n* = 17)	*P*-value

	Median (IQR) / *n* (%)	Median (IQR) / *n* (%)	
Age (in years)	59 (9)	55 (20)	0.595^a^

Gender			0.100^b^
Male	3 (60.0)	3 (17.6)	
Female	2 (40.0)	14 (82.4)	

Ethnicity			0.724^b^
Malay	3 (60.0)	13 (76.5)	
Chinese	1 (20.0)	3 (17.6)	
Indian	1 (20.0)	1 (5.9)	

Family history of CRC			>0.999^b^
Yes	0 (0)	2 (11.8)	
No	5 (100.0)	15 (88.2)	

Smoking status			>0.999^b^
Yes	0 (0)	2 (11.8)	
No	5 (100.0)	15 (88.2)	

Diabetes status			> 0.999^b^
Yes	1 (20.0)	5 (29.4)	
No	4 (80.0)	12 (70.6)	

BMI (kg/m^2^)	29.05 (11.81)	27.63 (6.44)	0.880

Notes:

+ Subjects with CRC was excluded from the analyses;

a Exact version of Mann-Whitney test;

b Fisher’s exact test;

IQR = interquartile range; CRC = colorectal cancer; BMI = body mass index

**Table 3:** Comparisons of pre and post-CRC awareness programme knowledge scores about CRC risk factors and signs and symptoms among study participants (*n* = 190)

CRC knowledge domains		Mean (SD)	Mean difference* (95% CI)	*P*-value
CRC risk factors	Pre-CRC awareness programme	30.92 (3.10)	−1.00 (−2.13, 0.16)	0.090
	Post-CRC awareness programme	29.92 (2.94)		

CRC signs and symptoms	Pre-CRC awareness programme	4.36 (3.10)	−1.60 (−2.10, −1.10)	**<0.001**
	Post-CRC awareness programme	2.76 (2.94)		

Notes:

* Post-CRC – Pre-CRC;

SD = standard deviation; CI = confidence interval; CRC = colorectal cancer. All statistical test assumptions for paired *t*-test procedures were met: Shapiro-Wilks *p*-values for normality of the differences between the pre and post-CRC awareness programme scores = 0.083 (CRC risk factors); 0.103 (CRC signs and symptoms); Fisher’s coefficient of skewness = 0.00 (CRC risk factors); 2.43 (CRC signs and symptoms)

**Figure:**
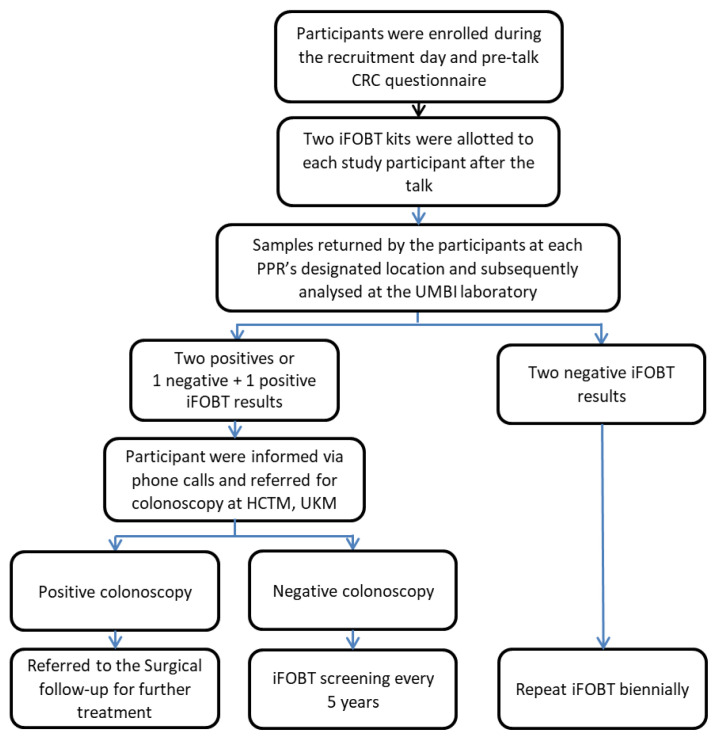
Flowchart of particiapant recruitment, enrollment, and follow-up in the CRC screening program

## Appendix 2

**Table:** Detailed breakdown of participant recruitment stratified by PPR locations (*n* = 321)

No.	Location of recruitments	No. of participants	No. returned kit	iFOBT result		Completed endoscopy
				Positive	Negative	
1	PPR Desa Tun Razak, 14/8/22	73	58 (79.5%)	14 (24.1%)	44	4
2	PPR Flat Sri Kota, 18/9/22	57	52 (91.2%)	21 (40.4%)	31	4
3	PPR Sri Johor, 15/10/22	28	19 (67.9%)	6 (31.6%)	13	1
4	PPR Sri Sabah, 15/1/23	31	25 (80.6%)	3 (12.0%)	22	1
5	PPR Taman Mulia, 19/3/23	39	36 (92.3%)	13 (36.1%)	23	3
6	PPR Sri Labuan, 18/6/23	41	38 (92.7%)	7 (18.4%)	31	2
7	Karnival Madani: Sihat & Prihatin, 9/7/23	13	11 (84.6%)	7 (63.6%)	4	5
8	PPR Taman Ikan Emas, 23/7/23	39	28 (71.8%)	9 (32.1%)	23	3
	Total	321	267 (83.2%)	80 (30.0%)	187	23 (28.8%)

## Appendix 3

**Table:** Sociodemographic determinants of colorectal polyps detection in the iFOBT-positive participants (*n* = 266)+

Characteristics	Simple logistic regression		Multiple logistic regression*	

	Crude OR (95% CI)	*P*-value	Adjusted OR (95% CI)	*P*-value
Age (years)	1.038 (0.913, 1.180)	0.569	1.031 (0.890, 1.196)	0.682

Male	2.849 (0.469, 17.305)	0.255	5.892 (0.877, 39.584)	0.068

Ethnicity				
Malay	Reference category		Reference category	
Chinese	4 (0.398–40.158)	0.239	3.370 (0.285, 39.842)	0.335
Indian	2.1 (0.213–20.688)	0.525	4.628 (0.392, 54.717)	0.224
Others	0.000 (0.000, NA)	0.999	0.000 (0.000, NA)	>0.999

Family history of CRC (Yes)	0.000 (0.000, NA)	0.998	0.000 (0.000, NA)	0.998

Smoking status (Yes)	0.000 (0.000, NA)	0.997	0.000 (0.000, NA)	0.998

Diabetes status (Yes)	0.431 (0.048, 3.937)	0.459	0.302 (0.028, 3.195)	0.320

BMI (kg/m^2^) (continuous)	1.011 (0.918, 1.114)	0.822	1.011 (0.940, 1.087)	0.768

APCS Score groups				
Low risk	Reference category		Reference category	
Moderate-risk	1.179 (0.129, 10.876)	0.884	1.249 (0.130, 12.013)	0.847
High risk	0.000 (0.000, NA)	0.998	0.000 (0.000, NA)	0.998

Note:

+ CRC case (*n* = 1) was excluded;

* *p*-value (Hosmer-Lemeshow) = 0.981 (without APCS Score) and 0.769 (with APCS score, diabetes, race and BMI).

Percentage of correct classification = 98.1% (for both models: i) without APCS Score; and ii) with APCS scores, diabetes, race and BMI). AUC = 0.838 (95% CI 0.735, 0.940) for the model without APCS Score and 0.723 (95% 0.506, 0.940) for the model with APCS score, diabetes, race and BMI. No multicollinearity (all bivariate correlations between predictors < 0.70 based on inspections of correlation matrix) and significant statistical interactions (all *p* interactions > 0.05) exist; NA = infinity; OR = odds ratio; CI = confidence interval; CRC = colorectal cancer; BMI = body mass index; APCS = Asia Pacific Colorectal Screening

## Appendix 4

**Table:** Sociodemographic determinants of CRC detection in the iFOBT-positive participants (*n* = 262)+

Characteristics	Simple logistic regression		Multiple logistic regression^*^	

	Crude OR (95% CI)	*P*-value	Adjusted OR (95% CI)	*P*-value
Age (years)	88,568.847 (0.000−.)	0.973	461,046.598 (0.000, NA)	0.973

Male	14,553,827.40 (0.000−.)	0.995	0.033 (0.000, NA)	> 0.999

Ethnicity				
Malay	Reference category		Reference category	
Chinese	0.000 (0.000, NA	0.999	337,609,874.46 (0, NA)	0.999
Indian	0.000 (0.000, NA)	0.998	0.09 (0, NA)	0.999
Others	0.000 (0.000, NA)	> 0.999	0.030 (0, NA)	> 0.999

Family history of CRC (Yes)	0.000 (0.000, NA)	0.999	15.350 (0, NA)	> 0.999

Smoking status (Yes)	41,422,431.749 (0.000, NA)	0.995	8.197 × 10^14^ (0, NA)	0.997

Diabetes status (Yes)	17,370,697 (0.000, NA)	0.996	286,594.174 (0, NA)	0.994

BMI (kg/m^2^) (continuous)	13,926,507.26 (0.000−.)	> 0.999	1.448 (0, NA)	0.998

APCS Score group				
Low risk	Reference category		Reference category	
Moderate-risk	1.000 (0, NA)	> 0.999	0.420 (0.000, NA)	> 0.999
High risk	38,463,686.124	0.998	11,920,308.986 (0.000, NA)	0.997

Notes:

+ Cases with polyps (*n* = 5) were excluded from the analyses;

** *P*-value (Hosmer-Lemeshow) > 0.999 [for both models i) with individual predictors without the APCS score groups; and ii) with APCS score, diabetes, race and BMI];

Percentage of correct classification = 100% and 99.6% for models: i) with individual predictors without APCS score group; and ii) with APCS score group, diabetes, race and BMI, respectively; AUC = 1.000 (95% CI 1.000, 1.000) indicating complete separation issue for the model with the individual predictors without the APCS score group and 0.976 (95% 0.958, 0.995) for the model with APCS score, diabetes, race and BMI; No multicollinearity (all bivariate correlations between predictors < 0.70 based on inspections of correlation matrix) and significant statistical interactions (all *p* interactions > 0.05) exist; NA = infinitely large or small (reaching 0); OR = odds ratio; CI = confidence interval; CRC = colorectal cancer; BMI = body mass index; APCS = Asia Pacific Colorectal Screening
